# Mechanisms Underlying CD4+ Treg Immune Regulation in the Adult: From Experiments to Models

**DOI:** 10.3389/fimmu.2013.00378

**Published:** 2013-11-18

**Authors:** Marta Caridade, Luis Graca, Ruy M. Ribeiro

**Affiliations:** ^1^Instituto de Medicina Molecular, Faculdade de Medicina da Universidade de Lisboa, Lisbon, Portugal; ^2^Instituto Gulbenkian de Ciência, Oeiras, Portugal; ^3^Theoretical Biology and Biophysics, Los Alamos National Laboratory, Los Alamos, NM, USA

**Keywords:** Tregs, mathematical models, CD4-blockade, regulation, tolerance

## Abstract

To maintain immunological balance the organism has to be tolerant to self while remaining competent to mount an effective immune response against third-party antigens. An important mechanism of this immune regulation involves the action of regulatory T-cell (Tregs). In this mini-review, we discuss some of the known and proposed mechanisms by which Tregs exert their influence in the context of immune regulation, and the contribution of mathematical modeling for these mechanistic studies. These models explore the mechanisms of action of regulatory T cells, and include hypotheses of multiple signals, delivered through simultaneous antigen-presenting cell (APC) conjugation; interaction of feedback loops between APC, Tregs, and effector cells; or production of specific cytokines that act on effector cells. As the field matures, and competing models are winnowed out, it is likely that we will be able to quantify how tolerance-inducing strategies, such as CD4-blockade, affect T-cell dynamics and what mechanisms explain the observed behavior of T-cell based tolerance.

## Introduction

Immunological tolerance can be defined as the state of unresponsiveness to an antigen, following prior contact with that antigen, where the host remains competent to mount an effective immune response against third-party antigens. Accomplishing therapeutic induced tolerance has been one of the major goals of immunology ever since the pioneering work of Medawar and colleagues (1).

There is a need to keep a balance between aggressive cells and cells that maintain tolerance to self. On occasions this balance can be disrupted originating either autoimmunity, when mechanisms leading to self-tolerance fail, or immunodeficiency and susceptibility to infection when the immune system is not able to mount a proper immune response. Usually, however, the immune system shows a significant capacity for self-tolerance, in spite of its equally efficient performance in the protection from foreign microbes. The ability to orchestrate protective immune responses is also the major hurdle impeding successful transplantation therapies and hinders the efficacy of therapeutic administration of foreign proteins and genes.

Random rearrangement of T-cell receptors (TCR) during cellular maturation leads to T cells that will recognize self-antigens. It was a long held assumption that central tolerance, by means of negative selection of autoreactive lymphocyte clones, could on its own account for the establishment of self-tolerance. Without such a censoring mechanism these autoreactive cells could eventually lead to autoimmune disease. Indeed thymocytes must survive the process of negative selection, which eliminates cells whose TCRs bind too avidly to self-antigens (2–4). The apoptosis of these thymocytes will prevent migration of autoreactive T cells to the periphery and prevent autoimmunity. Conversely, absence of negative selecting self-peptide-MHC complexes in the thymic medulla leads to an increase in mature autoreactive T cells (5, 6).

However, not all self-antigens are presented in the thymus, and some developing autoreactive T cells never encounter their antigens, eventually migrating to the periphery. Thus, although central tolerance contributes to the deletion of a large number of potentially autoreactive T cells, some autoreactive clones can be found in the periphery of healthy individuals (7, 8). There are, therefore, mechanisms that operate in the periphery (i.e., outside the thymus) to establish self-tolerance toward autoreactive clones that escape thymic negative selection.

Initially, one such mechanism was thought to be mediated by T-cell anergy, described as the functional state in which T cells remain viable but unable to respond to optimal stimulation through the TCR and co-stimulatory ligands (9), i.e., unable to proliferate or to produce interleukin-2 (IL-2) (10, 11). The first observation of anergy was made with purified human CD4^+^ T cells stimulated with large quantities of peptide antigens (10). It was noted that after antigen stimulation there was down-regulation of TCR and this was associated with the molecular mechanism for anergy (12). Subsequent studies with mouse CD4^+^ T cells suggested that occupancy of the TCR without any other signals was responsible for the induction of this state (13, 14).

Interestingly, anergic T cells were capable of suppressing proliferation of naïve T cells *in vitro* (15) and *in vivo* (16). In addition, anergic T cells have been shown to inhibit the antigen-presenting function and survival of dendritic cells (17). These and other observations led to the proposal of the “civil service model” (18), postulating that antigen-specific unresponsive cells can interfere with the generation of help by co-localizing with other T cells and competing for elements in the microenvironment (such as adhesion molecules or cytokines).

However, it was not clear how T cells would become anergic *in vivo*, and whether such mechanism was enough to maintain tolerance. More recently, a specific T cell subset, termed regulatory T (Treg) cells, gained prominence as being a key mechanism maintaining peripheral self-tolerance (19, 20). With hindsight, it is likely that many of the features of anergic T cells are a consequence of Treg function.

## Regulatory T Cells

In 1995 Sakaguchi et al. (19) showed that depletion of a minor population of CD4^+^ T cells constitutively expressing CD25 [IL-2 receptor α-chain (IL-2Rα)] led to the generation of a spectrum of autoimmune diseases when transferred to immune-compromised recipients. In addition, the co-transfer of CD25^+^ T cells prevented the pathology.

Based on this CD25 marker, a population of natural (thymus-derived) regulatory T cells was identified in the resting immune system, both in mice and in humans (21). Subsequent studies showed that these cells express forkhead box transcription factor 3 (Foxp3) and this finding led to the definite establishment of a Treg subset (22–24). There is now abundant evidence that these regulatory T cells are actively engaged in the maintenance of self-tolerance (25). Furthermore, depletion of Foxp3^+^ Tregs originates fatal multi-organ autoimmunity. The phenotype of this disease is virtually indistinguishable from the IPEX syndrome, caused by Foxp3 mutations in humans and equivalent to the Scurfy phenotype in mice (26–28).

### Thymic treg cells

The Treg cells that develop in the thymus, first described as naturally occurring regulatory T cells (nTregs) appear to be selected for self-antigen/MHC expressed by thymic epithelial cells (29, 30), in a process that requires TCR triggering in the presence of co-stimulation (31, 32), but dispenses TGF-β and IL-2 (33, 34). Early studies with Treg cells showed that these cells express CD25, CD5, and cytotoxic T lymphocyte antigen 4 (CTLA-4), which are all induced upon TCR stimulation (19).

In the periphery, nTregs represent around 6–10% of the overall CD4^+^ T-cell population. In order to be sustained they need continuous TCR triggering and co-stimulation in the presence of IL-2 (35–37), making IL-2 essential for natural Treg pool maintenance in the periphery (38). Comparative analysis of polyclonal TCR repertoires showed that TCR sequences from Treg cells were of broader variety and only partially overlapping with the ones from non-Treg cells (39). Some studies have shown that antigen-specific Treg cells are more potent at suppressing the induction of autoimmune disease than polyclonal populations (40). However, other studies have also shown that polyclonal Tregs are able to suppress independently of their specificity (41). Thus, Tregs with one antigen-specificity can suppress effector cells with many other antigen-specificities by bystander suppression. Moreover, transplantation studies have shown that Tregs can display a phenomenon called “linked suppression,” where they can be activated in an antigen-specific manner, and subsequently suppress responses to unrelated antigens presented by the same cells (42). Tregs show a third property called infectious tolerance by which one population of Treg cells creates a regulatory milieu that promotes the outgrowth of a new population of Treg cells with antigen-specificities distinct from those of the original population, as long as the new antigen is present in the same tissue as the antigen recognized by the original Treg cell (43–45).

### Peripheral treg induction

Besides nTreg, of thymic origin, it has become apparent that induced regulatory T cells (iTreg) also exist in the periphery (46, 47). After the discovery of the key role for Foxp3 in Tregs, it was demonstrated that it was possible for non-Treg cells to acquire both Foxp3 and the regulatory functions associated with it, therefore becoming Treg cells themselves (46, 48, 49).

It is likely that peripheral induction of iTreg occurs in response to non-self antigens like food, allergens, and commensal bacteria (39). Early evidence for *in vivo* peripheral conversion was derived from adoptive cell transfer experiments in which polyclonal CD4^+^ CD25^−^ naïve T cells were injected into lymphopenic mice or mice containing a monoclonal T cell repertoire devoid of nTregs, or when tolerance was imposed on monoclonal populations without Treg cells (49–51). In these conditions, homeostatic proliferation of the donor cells could be observed and part of the donor cell population became CD25^+^CTLA-4^+^GITR^+^Foxp3^+^ and acquired suppressive activity. Additionally, when congenitally marked CD4^+^ CD25^−^ T cells were transferred to WT hosts, 10% of those converted into CD4^+^ CD25^+^ Foxp3^+^ T cells, within 6 weeks (52).

It was first shown *in vitro* that TCR activation in the presence of TGF-β would lead to Treg conversion (53). Subsequent studies supported this observation and demonstrated that iTreg conversion could be greatly enhanced by suboptimal TCR signals or a combination of strong TCR signals with high doses of TGF-β (47, 53–57). *In vivo* it is possible to induce oral tolerance by giving the antigen in the drinking water (58), or to induce transplantation tolerance using non-depleting anti-CD4 at the time of transplantation (48, 59). In both cases, tolerance induction is accompanied by induction of Foxp3^+^ cells, in a process that requires TGF-β. In addition to these, many other factors influence the induction of Tregs both *in vitro* and *in vivo*, such as the co-stimulation environment, the strength of TCR signaling, mTOR inhibition with rapamycin, and low levels of essential amino-acids (44, 57, 60–69).

### Mechanisms of action of treg cells

In spite of intensive study of Tregs and their properties, the specific mechanisms by which they control immune responses are still not fully understood. There are several proposed mechanisms with experimental support, but it is likely that no single mechanism is responsible for the full range of biological phenomena involving Tregs (70). And it is also likely that in different milieu distinct mechanisms and even alternative subsets of regulatory cells are involved in tuning the immune response (71).

**Figure 1:**
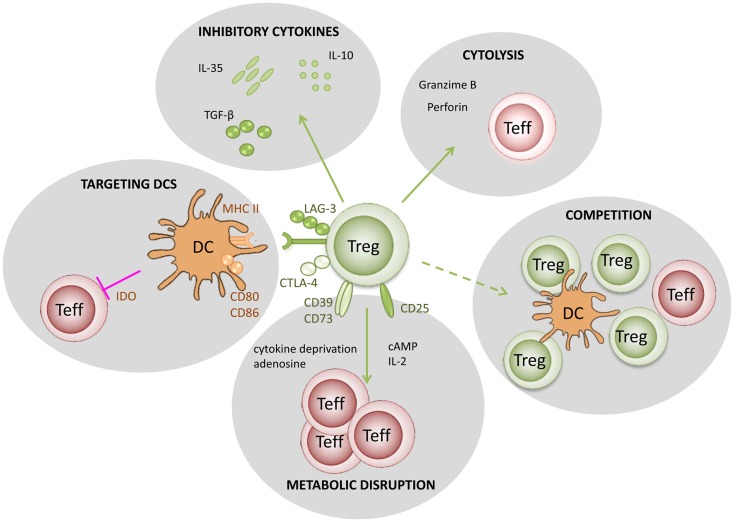
**Putative mechanisms used by regulatory T cells**. (1) Targeting DCs – modulation of antigen-presenting cell activity through Treg engagement of co-stimulatory receptors on the DC surface, leading to weak or abrogated signals to naïve/effector T cells; (2) Metabolic disruption – includes cytokine deprivation, cyclic AMP-mediated inhibition, and adenosine receptor (A2A)-mediated immunosuppression; (3) Competition – for critical cytokines, such as IL-2, or direct disruption of effector cell engagement with APCs; (4) Cytolysis – direct cytotoxic effect through the production of Granzyme B and Perforin and consequent apoptosis of effector T cells or APCs; (5) Production of inhibitory cytokines – including IL-10, IL-35, and TGF-β.

In Figure 1, we summarize five putative mechanisms of Treg function: (i) modulation of antigen-presenting cell (APC) activity through Treg engagement of co-stimulatory receptors on the surface of APC, leading to weak or abrogated signals from APC to naive/effector cells; (ii) Treg secretion of cytokines, such as IL10 and TGFβ, suppressing the activity of effector cells and APC; (iii) under certain circumstances, Tregs could have a direct cytotoxic effect, through the production of perforin/granzyme and induction of apoptosis in effector cells; (iv) Tregs may also cause metabolic disruption, for example stimulating APCs to produce enzymes that consume essential amino-acids, preventing naive/effector cell proliferation, and in the presence of TGFβ may induce the expression of Foxp3 in naive cells (i.e., they become Tregs); (v) Tregs could also compete with effectors cells for APC signals or cytokines, such as IL2.

There is mounting evidence [reviewed in (72)] that Treg cells exert their effects on different cell types, including CD4^+^ and CD8^+^ T cells, B cells, natural killer T cells (NKT), and DCs (70). The action of Tregs can be mediated by secretion of immunosuppressive cytokines, such as IL-10, TGF-β, IL-35, and galectin-1 (72) or by cell-dependent mechanisms through molecules such as GITR, CTLA-4, CD39, CD73, and LAG-3 (70). The spectrum of effect of Tregs on their targets goes from modifying the functional properties of other immune cells, such as down-regulating transcription of IL-2 (70, 71, 73), and other important growth factors; to actually killing those cells through granzyme B and perforin (70, 73–77). For example, there is evidence that Tregs can kill both immature and mature DCs (74).

Furthermore, Tregs may convert APCs to become themselves immunosuppressive (78). It has also been proposed that Tregs act by competing with other cells for growth factors, particularly IL-2 (79, 80). One possible outcome of these interactions is that other cells become themselves Foxp3^+^ regulatory cells (45).

These and other suppressive mechanisms may be operational depending on the microenvironment, biological context, and immune response. For instance, IL-10 producing cells are more abundant in lamina propria (81, 82) and perforin or granzyme expressing Tregs are predominant in tumor environments (83).

## Mathematical Modeling

Due to the complexity of the mechanisms and interactions involved in the processes of immune tolerance, mathematical modeling has been used as a tool to explore different conceptual frameworks of immunological tolerance. Many studies have analyzed the dynamics of thymocyte development, with positive and negative selection, as a mechanism of central tolerance (84–91).

Many other studies have focused on modeling the putative mechanisms of Treg suppression in the periphery. In these models, typically the dynamics of Tregs, effector cells, and APCs are studied to find the interaction mechanisms in the model that qualitatively reflect the experimental knowledge. For the purposes of this review, we can divide the models proposed in three categories, although there is overlap between these in some studies: (i) models that analyze different putative mechanisms of action of Tregs (Table 1); (ii) models that analyze the effects of Tregs on different processes, such as the immune response to pathogens and tumors, or in allergy; and (iii) models that study the maintenance of Tregs (homeostasis).

**Table 1:** **Summary of mechanistic models of Treg action**.

Cell populations considered	Mechanisms of regulation of immune response	Some properties of the model	Reference
APC, Treg, Teff, and Treg, Teff conjugates on APC	Competition for activation on APC Tregs inhibit Teff on same conjugate Treg maintenance is dependent on Teff	Treg inhibit growth of Teff Treg induce Teff to become Treg	(92–94, 96)
No explicit APC dynamics			
As above plus IL2	Competition for IL2	Non-local interactions	(97, 98)
	Tregs condition APC	Model used to study IL2-based therapies	
APC and Ag dynamics	Tregs directly suppress Teff (specifically and bystander)	Bystander effects are important	(99)
APC maturation	Tregs suppress APC maturation	Direct suppression was more effective	
T cells are activated into Treg or Teff by APC stimulation	
Antigen	Tp become Treg by interaction with resting APC	Strength of antigen stimulus is crucial in defining whether system is in tolerant or non-tolerant state	(100)
Immature APC, resting APC, activated APC	Tp become Teff by interaction with activated APC Teff activates APC Treg induces activated APC to rest	
Precursor T cells (Tp), Teff, Treg		
Stochastic model of TCR triggering for T cells (both thymus and periphery)	Different thresholds for activation vs. anergy, with or without co-stimulation	Self-reactive cells in periphery are controlled by a mechanism of reversible anergy	(103)
T cells with tunable activation thresholds	Model for integration of signals in successive encounters with APC	Exhibits self-tolerance	(102)
		“More cells should lead to less anergy,” which is not seen in adoptive transfer experiments	
Inactive and active Treg and Teff	Tregs consume IL2	Strength of antigen stimulation (for Treg and Teff) defines relative levels of those two populations	(107–109)
IL2 for Teff proliferation, also helps Treg proliferate	Treg inhibit Teff (from active to inactive) proportionally to Treg numbers	
Cytokine (e.g., IL7) for Treg homeostasis	
APC with different antigens Teff of multiple specificities Tregs of multiple specificities	Cells interact with extensive cross-reactivity, but different avidities	Effector functions are the outcome of individual cellular decisions (based on cross-reactivity)	(111)
		A threshold of conjugation time can be identified that permits self/non-self discrimination	

*Comparison of some of the models for Treg action discussed in the text. The model by Kim et al. (110) is too complex to fit in this summary table*.

### Models of the mechanisms of treg action

An early model explicitly considering Tregs was developed by León and collaborators (92). They considered cross-regulation, where simultaneous conjugation of a Treg and an effector cell on the same APC can suppress effector function (92, 93). In this model, regulation could be due to competition for conjugation sites on the APC, or through inhibitory signals delivered to effector cells on the same APC, or by inducing conversion of effector cells to a regulatory phenotype. This model is developed and analyzed in detail in several subsequent publications (93–95), and it is reviewed in Carneiro et al. (96). Recently the model was expanded to study the dual effect of IL2 in promoting immunity and tolerance (97, 98). Some authors considered in more detail the dynamics of antigen and APCs, and compared mechanisms where regulatory T cells suppress APCs function or maturation with models where Tregs act directly on effector T cells (99). Other models along these lines included the processes of APC maturation and the differentiation of T cells into regulatory or effector phenotypes (100), following a previous proposal for this interaction (101). Interestingly, in these models, survival or proliferation of Tregs is dependent on feedback from effector T-cells, which is in part responsible for the bi-stability observed that is interpreted as states of tolerance or immunity.

Another mechanism of peripheral tolerance modeled by several authors involves anergy of effector cells (102, 103). This anergy can be achieved by tuning the threshold for activation, for example through repeated encounter with antigen or APC (102–106), or through modulation by Tregs. Carneiro et al. compared this mechanism with their previous model of cross-regulation discussed above (102). Another model that also explores thresholds for activation, but based on effector T-cell population response was studied by Burroughs et al. (107, 108). In this model the relative levels of Tregs and effector T cells depend on the respective strength of stimulation by antigen, which can be modulated by IL-2 – this model is reviewed in (109).

Typically these models consider a limited number of cell populations (3–6) and analyze one mechanism at a time. However, Kim et al. proposed a detailed model including dozens of cell populations, with a spatial component (tissue and lymph node), and considered multiple mechanisms of Treg action simultaneously (110). At the other end of the spectrum, Abreu et al. proposed a model where regulation of the immune system was simply based on cross-recognition of multiple antigens by the same cell, whether it is an effector, a regulatory, or an APC (111).

### Models of the effect of tregs on the immune response

The studies discussed so far are mainly concerned with the mechanisms defining the interactions of Tregs and effector cells, often looking for steady states where one or the other population dominates, interpreted as tolerance or autoimmune states. Other models analyze the effects of the existence of Tregs on different processes.

Many of these models explore the system level effects of Treg failure and the potential development of autoimmunity. One of the first models to study this was by León et al. (112), where they analyzed the relationship between infections and autoimmunity in general. More recent studies analyzed specific autoimmune conditions, such as multiple sclerosis (113, 114) and inflammatory bowel disease (115). Grosse et al. analyzed the balance of Th1 vs. Th2 type responses and their control by Tregs in the context of allergies, with the objective of analyzing immunotherapy protocols (116). Another study looking at immunotherapy protocols, in this case modulation of IL2 therapy, used a mathematical model of helper, regulatory, and memory CD4^+^ T cells (98). These studies are mostly theoretical. However, one report described an interesting experimental study of mice injected with tolerogenic or control peptides and followed for 16 days, with serial measurements of different T-cell subsets. These data were then analyzed with a mathematical model (117).

Some studies have analyzed the interplay between infections and regulation of the immune response. One of these modeled the immune decision between attacking or not a given antigen, based on the network of interactions between Tregs, Th17 cells, and growing levels of antigen (as in the case of a pathogen) (118). And a model analyzing the regulation of the immune response in early HIV infection, through the expansion of specific Tregs, was recently introduced (119). Finally, León et al. (120, 121) considered an expansion of their mechanistic model of Treg – T effector interactions to study the immune response against tumors, and their control or expansion.

### Models of treg homeostasis

An important question that has also been addressed by modeling studies is the maintenance of a healthy number of Tregs. Several studies included the possibility of the Treg population being maintained in the periphery in part by feedback from the effector cells (92, 99, 100). One such model (94) studied the effects of thymic output and positive/negative selection on proper balance between tolerance and immunity in the periphery, and concluded that repertoire selection plays an important role in maintaining that balance. Baltcheva et al. (122) developed a more detailed model to analyze the life-long dynamics of precursor and mature CD25^+^ T cells (Tregs) in humans, including thymic production, density-dependent homeostasis, and effector T-cell conversion.

## Conclusion

The field of regulatory T cells, although relatively recent, has had an explosion of knowledge driven by detailed experimental work (20, 65, 72, 123, 124). Indeed there are many more studies than we could possibly review or even allude to in this mini-review. However, the mechanistic details of this important function of the immune system are not completely elucidated (72). Many authors have developed mathematical models of the interactions between Tregs and effector cells to try to add to our understanding of these mechanisms. Still, there is a lack of true collaborations between experimental scientists and modelers in this field. Clearly, more progress would be possible if such integrated teams worked together, as has been the case in other areas of medicine, e.g., modeling of viral infections (125). As the field matures and competing models are winnowed out, it is likely that we will be able to quantify how tolerance-inducing strategies, such as CD4-blockade, affect T-cell dynamics, and what mechanisms explain the observed behavior of T-cell based tolerance.

## Conflict of Interest Statement

The authors declare that the research was conducted in the absence of any commercial or financial relationships that could be construed as a potential conflict of interest.
